# Immune Activation Following Vaccination of *Streptococcus iniae* Bacterin in Asian Seabass (*Lates calcarifer*, Bloch 1790)

**DOI:** 10.3390/vaccines11020351

**Published:** 2023-02-03

**Authors:** Pornpawit Tanpichai, Surachart Chaweepack, Saengchan Senapin, Patharapol Piamsomboon, Janenuj Wongtavatchai

**Affiliations:** 1 Department of Veterinary Medicine, Faculty of Veterinary Science, Chulalongkorn University, Bangkok 10330, Thailand; 2 Chanthaburi Coastal Aquaculture Research and Development Center, Chanthaburi 22000, Thailand; 3 Fish Health Platform, Center of Excellence for Shrimp Molecular Biology and Biotechnology (Centex Shrimp), Faculty of Science, Mahidol University, Bangkok 10400, Thailand; 4 National Center for Genetic Engineering and Biotechnology (BIOTEC), National Science and Technology Development Agency (NSTDA), Pathum Thani 12120, Thailand

**Keywords:** aquaculture, Asian seabass, bacterin, ELISA, formalin-killed vaccine, qPCR, streptococcosis

## Abstract

Juvenile Asian seabass (*Lates calcarifer*) (body weight 10 ± 0.7 g) were intraperitoneally injected with 10^12^ CFU fish^−1^ of formalin-killed *Streptococcus iniae*. The protective efficacy of the vaccine on survival and infection rate was assessed upon challenge at 4, 8, 12, 20, and 28 weeks post-vaccination. The results revealed that the challenged vaccinated fish showed no mortality at all time points, and the control fish presented 10–43.33% mortality. The infection rate at 2 weeks post-challenge was 0–13.33% in the vaccinated fish and 30–82.35% in the control group. At 8 weeks post-vaccination, the vaccinated fish showed comparable ELISA antibody levels with the control; however, the antibody levels of the vaccinated fish increased significantly after the challenge (*p* < 0.05), suggesting the presence of an adaptive response. Innate immune genes, including MHC I, MHC II, IL-1β, IL-4/13B, and IL-10, were significantly upregulated at 12 h post-challenge in the vaccinated fish but not in the control. In summary, vaccination with *S. iniae* bacterin provided substantial protection by stimulating the innate and specific immune responses of Asian seabass against *S. iniae* infection.

## 1. Introduction

Asian seabass or Barramundi (*Lates calcarifer*) is an important economic fish species cultured in Australia and southeast Asia, including Malaysia, Singapore, and Thailand. This euryhaline fish is suitable for culture in seawater, brackish water, and freshwater. In 2020, the amount of Asian seabass produced in Thailand was 45,415.25 tons, yielding a revenue of 450 million baht [1]. This increase in production requires high stocking density, leading to poor water quality and deteriorated fish health. Several diseases have emerged as a major constraint in Asian seabass culture, including streptococcosis caused by *Streptococcus iniae* [2]. This Gram-positive bacterium is associated with the large-scale mortality of Asian seabass culture in marine and freshwater. An infected fish shows clinical signs, such as a loss of equilibrium, erratic swimming, unilateral or bilateral exophthalmia, corneal opacity, darkening skin, and hemorrhaging at the fins [3]. The outbreak was first recorded in 1999 from marine-cultured seabass farms in Australia [4] and was subsequently reported in several countries, including Singapore [5], Vietnam [6], Iran [7], and Israel [8]. In Thailand, *S. iniae* was reported as a cause of mass mortality in marine and freshwater seabass culture [3,9,10].

The treatment of bacterial infection requires the use of antimicrobial drugs, which are usually accompanied by drug resistance and drug residue problems, especially in grown-out fish for human consumption. Vaccination is an alternative method to reduce the severity of disease and the imprudent use of antimicrobial drugs by stimulating fish immunity against the disease. In aquaculture, inactivated or killed vaccines are commonly utilized due to their stability in the field and lower production cost compared with other types of vaccine [11]. Although inactivated vaccines often show less efficacy against viral or intracellular bacterial infection [11], the bacterin provides sufficient protection against several bacterial infections, such as vibriosis [12,13], furunculosis [14], and streptococcosis [15]. Inactivated vaccines against *S. iniae* in fish have been widely studied in several species, such as Nile tilapia (*Oreochromis niloticus*) [15,16,17], grouper (*Epinephelus coioides*) [18], zebrafish (*Danio rerio*) [19], rainbow trout (*Oncorhynchus mykiss*) [20,21,22], and Asian seabass [23,24]. Studies in Asian seabass showed the efficacy of inactivated vaccines against experimental challenge at 60 and 79 days post-vaccination and the increasing IgM response to vaccination [23,24]. Considering that commercial *S. iniae* vaccine for Asian seabass is not available in Thailand, our study aimed to evaluate the efficacy of inactivated vaccines using several parameters, including mortality rate and infection rate after challenge, antibody levels, and expression levels of immune-related genes. 

## 2. Materials and Methods

### 2.1. Experimental Fish and Husbandry

A total of 370 healthy juvenile Asian seabass with average body weight of 10 ± 0.7 g were stocked at the Chanthaburi Coastal Aquaculture Research and Development Center, Department of Fisheries, Ministry of Agriculture, and Cooperatives, Chanthaburi, Thailand. The fish were acclimatized in 500 L plastic tanks with 20 fish per tank supplied with filtered seawater (35 ppt salinity) and full aeration for 2 weeks prior to the experiment. The fish were fed with commercial diet (Profeed^®^, Thai Union Feedmill, Samut Sakhon, Thailand) containing 42% crude protein twice a day at 1% of bodyweight. Water temperature, pH, unionized ammonia, and nitrite were maintained at 26 °C–32 °C, 7.5–8.5, <1 mg L^−1^, and <0.01 mg L^−1^, respectively. Ten fish were randomly collected for prestudy health examination, including ectoparasite examination and bacterial culture from anterior kidney, to ensure that the fish were clinically healthy. This experiment was approved by the animal use ethics protocol of the Chulalongkorn University Animal Care and Use Committee (CU-ACUC: Approval No. 2231042). 

### 2.2. Preparation of Formalin-Killed S. iniae Bacterin

For vaccine preparation, the protocol described by Jantrakajorn et al. [25] was adopted. Streptococcal isolate was obtained from the collection at the Department of Veterinary Medicine, Faculty of Veterinary Science, Chulalongkorn University. The isolate was recovered from −70 °C onto tryptic soy agar (TSA; Oxoid, Basingstoke, UK) and incubated at 33 °C for 24 h. The bacteria were resuspended in sterile normal saline solution (NSS) to yield a cell density of 0.5 McFarland (10^8^ cells mL^−1^), inoculated in 100 mL of tryptic soy broth (Oxoid, Basingstoke, UK), and incubated at 33 °C for 12 h using a shaking incubator at 100 rpm (Sheldon MFG., Cornelius, OR, USA). A bacterial pellet was harvested by centrifugation at 6500× *g* for 20 min, treated with 3% formalin at 4 °C for 12 h, and centrifuged at 7500× *g* for 35 min. The cell pellet was washed twice with sterile NSS and adjusted to an optical density of 1.1 at 540 nm wavelength, equivalent to 1 × 10^13^ cells mL^−1^. The sterility of bacterins was determined as the lack of growth of any bacteria after being plated onto TSA and incubated at 33 °C for 48 h. 

### 2.3. Vaccination and Challenge Protocol

A total of 150 fish were intraperitoneally injected with 0.1 mL of 10^12^ CFU fish^−1^ of *S. iniae* bacterin, and the other set of fish was assigned to control group and injected with sterile normal saline solution (NSS). Ten fish from each group were challenged with 10^10^ CFU mL^−1^ intraperitoneal injection of *S. iniae* (0.1 mL per 10 g fish) at 4, 8, 12, 20, and 28 weeks post-vaccination in triplicate. The fish were observed for morbidity, feeding behavior, clinical signs, and mortality at 14 days after challenge. At termination date, all fish were euthanized with overdose anesthetic solution (Aquanes^®^, Better Pharma, Bangkok, Thailand) and aseptically dissected approaching an anterior kidney for bacterial culture on TSA. The plates were incubated at 25 °C–27 °C overnight. Kidney and spleen tissues of each fish were preserved in 70% ethanol for further PCR assay, which was applied in fish with negative result from bacterial culture. Infection rate was calculated as the number of fish positive for *S. iniae*, indicated by bacterial culture and PCR assay.

For PCR assay, genomic DNA was extracted using Nucleospin^®^ Tissue mini kit (Macherey-Nagel, Dueren, Germany) and replicated by PCR after mixing with DreamTaq PCR Master Mix (2X) (Thermo Fisher Scientific, Waltham, MA, USA) and PCR primers (SP1 5′-GAAAATAGGAAAGAGACGCAGTGTC-3′ and SP2 5′-CCTTATTTCCAGTCTTTCGACCTTC-3′) for a 377 bp fragment of the *S. iniae* 16S rRNA gene [26]. Thermal cycling conditions were as follows: initial denaturation at 95 °C for 3 min, followed by 35 cycles of 95 °C for 30 s, 60 °C for 30 s, and 72 °C for 60 s, and a final extension of 72 °C for 5 min. Finally, the amplified product was visualized in 1.5% agarose gel (Thermo Fisher Scientific, Waltham, MA, USA) using MUPID-exU^®^ Electrophoresis System (Takara Bio Inc., Shiga, Japan).

### 2.4. Enzyme-Linked Immunosorbent Assay (ELISA)

Blood samples were collected from vaccinated and control fish at 8 weeks post-vaccination and 1-week post-challenge survivors to evaluate specific antibody (IgM) response. The ELISA protocol followed that in Thu-Lan et al. [24]. In brief, the collected blood samples were centrifuged at 2000× *g* 4 °C for 12 min in a universal centrifuge (SIGMA Laborzentrifugen GmbH, Osterode am Harz, Germany) to collect serum. Flat-bottom microplate wells (Costar^®^, Corning Inc., Corning, NY, USA) were coated with 100 μL well^−1^ of 10^8^ CFU mL^−1^ *S. iniae* whole-cell antigen, incubated at 4 °C overnight, and washed three times with 1X PBS containing 0.05% Tween-20 (Amresco, Solon, OH, USA) (PBST). Serum samples were diluted with PBST containing 0.2% skimmed milk (PBSTM) at 1:1500 and incubated at 4 °C overnight. After being washed three times with PBST, anti-seabass IgM (Marine Leader, Bangkok, Thailand) [27] diluted with PBSTM (1:50) was overlayed and incubated for 2 h, followed by washing with PBST and adding commercial goat anti-mouse antibody horseradish peroxidase conjugate (Thermo Fisher Scientific, Waltham, MA, USA) diluted in PBSTM (1:3000) into each well for 1 h. After washing, 3, 3′, 5, 5′ -tetramethylbenzidine substrate (EMD Millipore Corp, Billerica, MA, USA) was added, and the wells were incubated for 15 min with gentle shaking. The reaction was stopped by adding 100 μL of 2 M H_2_SO_4_ into each well. Finally, a microplate reader (SpectraMax^®^, Molecular Devices, San Jose, CA, USA) was used to measure the absorbance at 450 nm.

### 2.5. Immune-Related Gene Expression

The expression of immune-related genes in challenged and nonchallenged fish groups (n = 15 per group) was evaluated. The fish were challenged at 4 weeks post-vaccination or sham vaccination (control), and tissues for immune gene analysis were collected at 12, 24, and 48 h after challenge. The nonchallenged fish were examined at 12, 24, and 48 h after vaccination or sham vaccination (n = 5 per time point).

#### 2.5.1. RNA Extraction and cDNA Synthesis

Spleen and kidney tissue from individual fish were pooled and allocated as one sample. The tissues were preserved in 1.5 mL microfuge tube containing Trizol^®^ (Invitrogen, Waltham, MA, USA) and kept refrigerated. RNA extraction was performed using commercial kit (Direct-zol™ RNA Miniprep, Zymo Research, Irvine, CA, USA). Homogenized samples were mixed with 200 μL of chloroform and incubated at room temperature for 5 min. The samples were then centrifuged at 12,000× *g* and 4 °C for 20 min to transfer the transparent aqueous phase, and the aqueous was mixed with 70% ethanol. The solution was added with RNA prewash, deoxyribonuclease I enzyme, RNA wash buffer, and ribonuclease free water. Lastly, the amount of RNA was calculated using Qubit^®^ RNA BR Assay Kit (Invitrogen, Waltham, MA, USA) and Qubit^®^ fluorometer (Invitrogen, Waltham, MA, USA). The quantity of RNA must exceed 110 ng μL^−1^ before proceeding to the converting step. Reverse-transcript polymerase chain reaction) was performed to convert RNA to cDNA. The extracted RNA was denatured at 65 °C for 10 min and further processed with ImProm-II™ Reverse Transcription System (Promega, Madison, WI, USA). The samples were generated at 42 °C for 90 min and 95 °C for 2 min.

#### 2.5.2. Quantitative Real-Time PCR (qPCR)

qPCR was utilized to assess the levels of immune-related genes using a CFX Connect Real-Time PCR System (Bio-Rad Laboratories, Munich, Germany). qPCR mixture was prepared as 14 μL of mixture containing 7.5 μL of PowerUp™ SYBR™ Green Master Mix (Kapa Biosystems, Hertfordshire, UK), 0.3 μL of ROX high (Kapa Biosystems, Hertfordshire, UK), 0.3 μL of per primer (Table 1), 1 μL of cDNA (100 ng), and 4.6 μL of nuclease free water. Thermal cycling conditions were as follows: initial denaturation at 95 °C for 2 min, followed by 40 cycles of 95 °C for 10 s, 60 °C for 10 s, and 72 °C for 10 s, and final extension of 72 °C for 5 min. Relative quantification was performed by comparing the levels of the target gene to an internal gene (ribosomal 18S rRNA), and the control sample was used as a calibrator. The internal gene was included in the analysis to correct for differences in total cDNA input among the samples. Quantitative assessment was based on threshold cycle (Ct), the cycle in which a statistically significant increase in fluorescence above the background signal detection is observed. The specificity and size of the qPCR products were confirmed by agarose gel electrophoresis. The results were expressed as mean (Table S1).

### 2.6. Data Analysis

R software (https://www.rstudio.com, accessed on 1 October 2022) was used for all statistical analysis. Shapiro test was applied to evaluate the normality of data. ANOVA with Tukey’s post-hoc test was employed to determine the significant differences of the parameters, including antibody level, and immune gene expression level, among groups. *p*-value of <0.05 was accepted as significant.

## 3. Results

### 3.1. Bacterial Challenge

**Figure 1:**
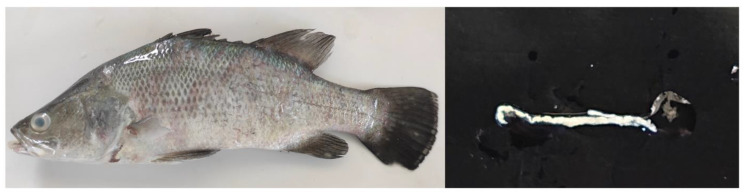
Juvenile Asian seabass intraperitoneally infected with *S. iniae* 10^10^ CFU mL^−1^ showing bilateral lens opacity and scale loss (**Left**). The whitish feces from the sick fish were observed in the experimental aquaria (**Right**).

The protective efficacy of the vaccine was evaluated by recording the cumulative mortality and infection rate of the fish at 2 weeks post-challenge. No mortality was observed in the challenged vaccinated fish at all time points. Meanwhile, the challenged control fish reached the highest mortality at 3 days post-challenge and showed a cumulative mortality of 43.33%, 10%, 26.66%, 40%, and 0% at 4, 8, 12, 20, and 28-weeks post-sham vaccination, respectively. In addition, the infected fish exhibited clinical signs, including a loss of appetite, bilateral lens opacity, scale loss, skin darkening, and whitish feces (Figure 1). The challenged vaccinated fish did not show any clinical sign except for a loss of appetite for 2–3 days post-challenge. The bacterial culture test in every dead fish showed a 100% positive result for *S. iniae*. Moreover, PCR assay revealed notably higher infection rate in the control survivors than in the vaccinated survivors. The infection rates at 4, 8, 12, 20, and 28 weeks post-vaccination were 0%, 13.33%, 10%, 0%, and 0% for the vaccinated survivors, respectively, and 82.35%, 37.03%, 68.18%, 77.78%, and 30% for the control survivors, respectively (Table 2).

### 3.2. IgM Antibody Response

**Figure 2:**
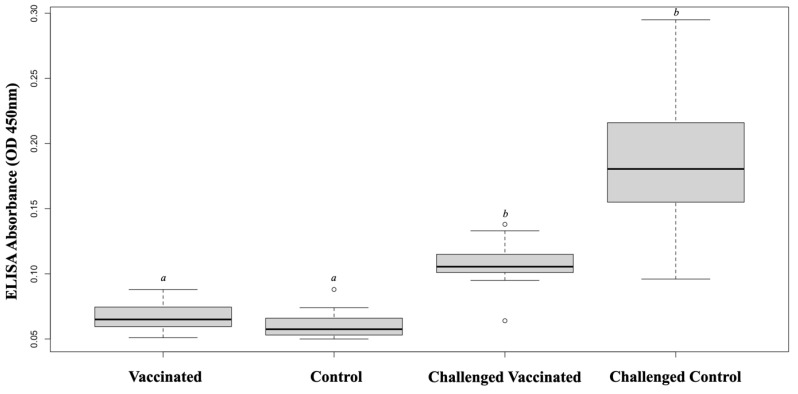
Serum antibody titers against *S. iniae* determined by ELISA at 8 weeks post-vaccination were not different between the vaccinated and control groups. Both groups showed significantly high antibody levels following a challenge with *S. iniae*. Significant differences (*p* < 0.05) are indicated with different letters.

ELISA was used to evaluate the specific antibody response in the vaccinated and control fish at before and after challenging. ELISA absorbance values (OD 450 nm) for sera (1:1500 dilution) were 0.067 ± 0.01 and 0.060 ± 0.01 in the vaccinated and control fish, respectively. However, significantly higher ELISA values were observed in the vaccinated and control survivors compared with those in the nonchallenged groups (*p* < 0.05). The antibody titers of challenged survivors were not different between the vaccinated and control groups (Figure 2).

### 3.3. Immune-Related Gene Expression

**Figure 3:**
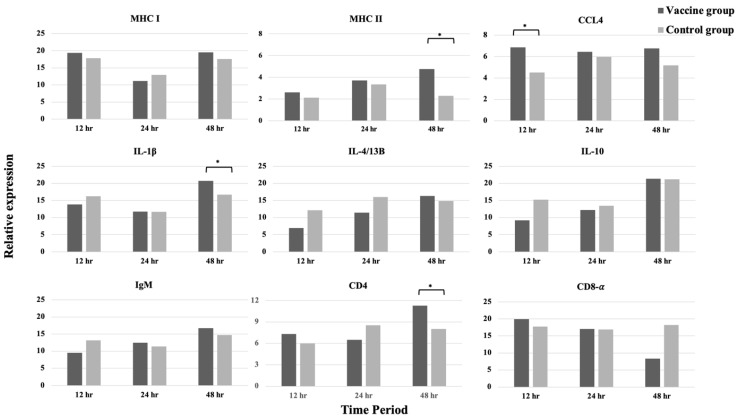
Expression profiles of immune-related genes in the vaccinated and control fish at 12, 24, and 48 h post-vaccination (* *p* < 0.05).

**Figure 4:**
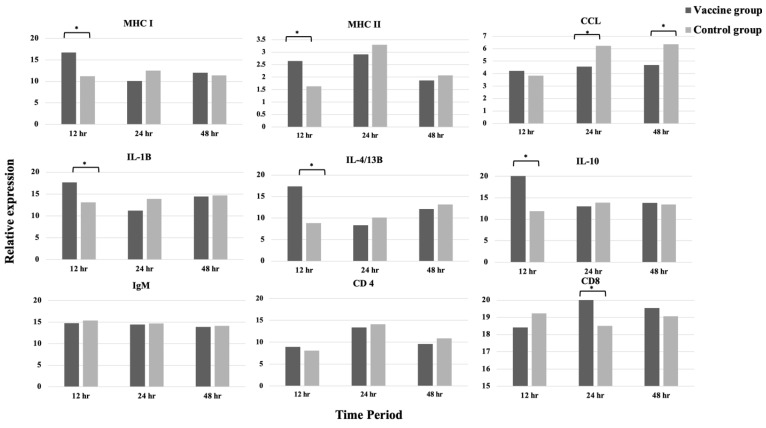
Expression profiles of immune-related genes in challenged vaccinated survivors and challenged control survivors at 12, 24, and 48 h post-challenge (* *p* < 0.05).

The vaccinated fish showed significantly high CCL4 expression at 12 h and MHC II, IL-1β, and CD4 expression at 48 h (*p* < 0.05) (Figure 3). At 4 weeks post-vaccination, the vaccinated fish showed higher expression levels of MHC I, MHC II, IL-1β, IL-4/13B, and IL-10 at 12 h post-challenge (*p* < 0.05) compared with the challenged control fish. Moreover, the CD8-α gene representing cytotoxic T cells was upregulated in the challenged vaccinated fish at 24 h after challenge but was not prominent in the challenged control (*p* < 0.05). On the contrary, CCL4 was upregulated in the challenged control but not in the challenged vaccinated fish at 24 and 48 h (*p* < 0.05) (Figure 4).

## 4. Discussion

Inactivated vaccines or bacterins against *S. iniae* have been used in rainbow trout (*O. mykiss*) since 1997 [18,22] and have been applied via several routes, including injection, oral, and immersion. Administration via injection is generally the major route to achieve a high efficacy of vaccines, primarily because the fish can receive appropriate antigen dosage [18,33]. Klesius et al. [15] reported that for inactivated vaccines, a homologous vaccine administered via the intraperitoneal route showed better efficacy than that given via the intramuscular route, with a significantly higher relative percentage of survival (45.6% from IP route and 17.7% from IM route) for tilapia (*O. niloticus*). Karami et al. Ref. [34] explained that the good efficacy of IP route was because the bioavailability of the antigens was 4.5 times higher via IP route than via IM route. Thus, we focused on inactivated vaccines given via the intraperitoneal route. 

In the challenge experiment, no mortality or morbidity were found among the vaccinated fish. Meanwhile, the control fish showed a 10–43.33% mortality rate with significant clinical illness and high infection rates at all time points. This finding indicated the effectiveness of *S. iniae* bacterin in protecting fish against disease. In addition, most of the vaccinated fish had no remnants of *S. iniae* at 2 weeks post-challenge, as indicated by their negative PCR result. Although some of the control fish survived, the presence of whitish feces may imply the pathological condition of their intestines that subsequently impaired their growth performance [35]. Moreover, the control survivors may become a reservoir for the pathogen because a high infection rate was prominent. Therefore, the vaccine may not only prevent mortality but also reduce disease spreading from infected individuals. Previous studies also reported the efficacy of inactivated vaccines against *S. iniae* in seabass. The vaccination provided 50% survivals following intraperitoneally challenge [23] and 70% survivals after 10^6^ CFU Fish^−1^ intraperitoneally challenge [24]. The *S. iniae* bacterin has also been investigated in other fish species, including rainbow trout [22], tilapia [15], hybrid striped bass (*Morone chrysops × M. saxatilis*) [36], grouper [18], and red seabream (*Pagrus major*) [37], with more than 80% survival rate recorded in the challenged vaccinated fish. Even though inactivated vaccine might stimulate a weaker immune response than other kinds of vaccine [11], it is cost-efficient, and sufficiently protects the fish from disease throughout the culture period. As shown in our study, the formalin-killed vaccine provides up to 7 months of protection against *S. iniae* infection.

The susceptibility to streptococcosis caused by *S. iniae* may be age-dependent. The vaccinated and control fish at 7 months post-vaccination (130 ± 12 g body weight) showed no mortality upon the challenge. Múzquiz et al. [38] suggested that large rainbow trout fish can eliminate the pathogen with no mortality because of their stronger immunity response compared with the younger fish. Even though some reports showed the mass mortality of Asian seabass in grown-out farm caused by streptococcosis [3,4,39], the severity of the outbreak may be associated with disease conditions, such as water quality, density of fish, and husbandry management. 

The specific antibody response against *S. iniae* remarkably increased in the challenged vaccinated and control survivors 1 week after challenging compared with that in the nonchallenged fish. Nevertheless, the challenged control survivors showed variations in their antibody levels, as indicated by high standard deviation due to each individual immune status. Similar to our study, the increasing antibody level of vaccinated fish after challenging was noted in Japanese flounder (*Paralichthys olivaceus*) receiving multivalent vaccine against *S. iniae*, *Edwardsiella tarda*, *Vibrio anguillarum*, and *Vibrio harveyi* [40]. In the present work, we observed serum antibody at 8 weeks post-vaccination because the size of the fish was suitable for blood collection. We also found a slightly higher but comparable amount of antibody titers in the vaccinated group relative to that in the control fish. However, once the fish immunity was stimulated by a challenge, the antibody levels increased immediately within 1 week. This finding may support the presence of immunoglobulin memory in the vaccinated population.

Several studies observed the alteration of immune-related genes following vaccination or challenge as a surrogate marker for protective immunity [41]. In our study, the vaccination of seabass with *S. iniae* bacterin activated the expression of immune genes in the pooled spleen and kidney samples. RT-PCR revealed the significant upregulation of IL-1β, MHC II, and CD4 genes in the fish at 48 h after vaccination. This phenomenon might have occurred due to the early immune response and inflammatory process in fish. The early increase in IL-1β as a pro-inflammatory cytokine induced a cascade of reactions, leading to inflammatory process, and migration of innate cellular immunity, such as neutrophils and macrophages [42,43]. MHC II is a member protein of antigen-presenting cells that interact with T cells for stimulating immune response [29]. The increased expression of these genes after vaccination might be the immunological basis of the vaccine [44]. Post-challenge, MHC I, MHC II, IL-1β, IL-4/13B, and IL-10 expression levels were significantly elevated in the vaccinated fish at 12 h, indicating the role of vaccine in stimulating the innate immune response to recognize and control the pathogen after exposure. Similar to MHC II, MHC I is also a member protein of antigen-presenting cells, and its expression might be increased upon macrophage activation. IL-4/13B is a pleiotropic cytokine secreted by T cells to regulate Th2 differentiation and mediate antibody response [43]; therefore, the increase in IL-4/13B in this study may be associated with B cell maturation and antibody production. The upregulation of IL-10, an anti-inflammatory cytokine, may serve as negative feedback to control the inflammation process following the challenge. A similar result was found in vaccinated flounder that received monovalent and divalent DNA vaccine against *S. iniae* and *V. anguillarum* and showed increasing levels of MHC I and II after challenge at 4 and 24 h, respectively [45,46]. Liu et al. [47] also showed the elevation of IL−1β after 24 h post-challenge from flounder immunized with DNA vaccine against *S. iniae*. Sivasankar et al. [48] observed the increased expression of IL−10 in the spleen of Asian seabass receiving inactivated vaccine against damselfish virus at 12 h to 48 h after challenge. Even though the change in gene expression level was not evident at post-vaccination compared with that at post-challenge, the potency of the vaccine in enhancing innate immune system was manifested by the prompt expression of many genes within 12 h after the pathogen invasion. Together with adaptive response, these quick responses may play an important role in keeping the fish alive. 

Adaptive immunity is the main point of interest in vaccine development. At the time of our observation, the expression of adaptive immune response genes, including IgM, CD4, and CD8-α, is not yet accepted as evidence. We detected their expression within 48 h after stimulation, which may not be an appropriate time point. The expression of IgM gene in European seabass (*Dicentrarchus labrax*) was not detected at 48 h following Noda virus challenge [49]. The earliest response of IgM gene was recorded at 72 h after exposure to betanodavirus in European seabass [32]. Similarly, Yang et al. [50] reported an increase in CD8-α gene expression in rainbow trout at 5 days post-challenge with *Yersinia ruckeri*. Although the detection of the expression of adaptive immune gene was unsuccessful in our study, evidence of an adaptive immune response was demonstrated with outstanding ELISA antibody titer detected from the challenged vaccinated fish.

## 5. Conclusions

The formalin-killed *S. iniae* vaccine in this study provides substantial protection against experimental challenge and causes no mortality and significantly low infection rate in vaccinated fish. Its immunoprotective efficacy is supported by the prompt response of innate and adaptive immunities as shown by the increased antibody titer and gene expression levels. This work calls attention to the importance of vaccine application in juvenile Asian seabass for protection against severe infectious diseases.

## Figures and Tables

**Table 1:** Primer sequences used for qPCR.

Genes	Target	Sequences (5′-3′)	References
Internal control	18S rRNA	F: TGGTTAATTCCGATAACGAACGA R: CGCCACTTGTCCCTCTAAGAA	Wang et al. [28]
Innate immune genes	MHC I	F: GGCTGTTTTTGCCGCTCTG R: GTGGACAGGTCTGGATAAAG	Silvaraj et al. [29]
	MHC II	F: GTTGGATACACTGAGTTTGG R: GTTGGATACACTGAGTTTGG	Mohd-Shaharuddin et al. [30]
	CCL4	F: TCCTCGTCTACTCTGTCTGT R: GACCTGCCACTGTCTTCAGC	Silvaraj et al. [29]
	IL-1β	F: ATCTGGAGGTGGTGGACAAA R: AGGGTGCTGATGTTCAAACC	Silvaraj et al. [29]
	IL-4/13B	F: TCATGAAGACGCAAATCTGATGT R: CGAGACAGGAGAACTCTTTCACACA	Buonocore et al. [31]
	IL-10	F: CGACCAGCTCAAGAGTGATG R: AGAGGCTGCATGGTTTCTGT	Silvaraj et al. [29]
Adaptive immune genes	IgM	F: GAGCTGCAGAAGGACAGTG R: TCAGACTGGCCTCACAGCT	Scapigliati et al. [32]
	CD4	F: GTGATAACGCTGAAGATCGAGCC R: GAGGTGTGTCATCTTCCGTTG	
	CD8-α	F: CTAAGATTCGGCAAAATAACTCGA R: GATGAGGAGTAGAAGAGAAGGCC	

**Table 2:** Mortality rate and infection of the vaccinated and control fish following a challenge at 4, 8, 12, 20, and 28 weeks post-vaccination.

Weeks Post-Vaccination (Bodyweight, g)	Group	Mortality (%)	Infection		
			Bacterial Culture	PCR	Total (%)
4 (10 ± 0.7)	Vaccine	0	0	0	0
	Control	13/30 (43.33)	13	1	14/17 (82.35)
8 (48 ± 3.5)	Vaccine	0	0	4	4/30 (13.33)
	Control	3/30 (10.00)	4	6	10/27 (37.03)
12 (70 ± 5.8)	Vaccine	0	1	2	3/30 (10.00)
	Control	8/30 (26.66)	12	3	15/22 (68.18)
20 (97 ± 9.4)	Vaccine	0	0	0	0
	Control	12/30 (40.00)	12	2	14/18 (77.78)
28 (130 ± 12.6)	Vaccine	0	0	0	0
	Control	0	0	9	9/30 (30.00)

## Data Availability

The data that support the findings of the study are available on request from the corresponding author.

## Supplementary Materials

The following supporting information can be downloaded at: https://www.mdpi.com/article/10.3390/vaccines11020351/s1, Table S1: The immune related gene expression assessed as mean of threshold cycle (Ct) value.
