# Supplementary material for: Immune Activation Following Vaccination of Streptococcus iniae Bacterin in Asian Seabass (Lates calcarifer, Bloch 1790)
Source: Vaccines (Basel). 2023 Feb 3;11(2):351. doi: 10.3390/vaccines11020351 (PMC9963699; doi:10.3390/vaccines11020351)
Supplement: Supplementary file 1 [file vaccines-11-00351-s001.zip › vaccines-2160215-supplementary.pdf]

Supplementary Table S1: The immune related gene expression assessed as mean of threshold cycle (Ct) value.

Gene	Time (hr)	Post-vaccination		Post-challenge	
		Vaccine	Control	Vaccine	Control
**MHC I**	12	19.375	17.805	16.728	11.200
	24	11.166	12.955	10.080	12.512
	48	19.498	17.607	12.015	11.428
**MHC II**	12	2.600	2.133	2.6475	1.630
	24	3.715	3.337	2.915	3.297
	48	4.767	2.294	1.868	2.065
**CCL 4**	12	6.860	4.525	4.218	3.827
	24	6.453	5.988	4.560	6.223
	48	6.778	5.188	4.690	6.347
**IL-1β**	12	13.895	16.273	17.687	13.130
	24	11.740	11.695	11.200	13.902
	48	20.740	16.753	14.462	14.660
**IL-4/13B**	12	6.956	12.178	17.380	8.805
	24	11.455	15.995	8.310	10.128
	48	16.307	14.838	12.083	13.177
**IL-10**	12	9.240	15.225	20.778	11.895
	24	12.224	13.460	12.988	13.865
	48	21.354	21.230	13.822	13.435
**IgM**	12	9.545	13.142	14.733	15.382

	24	12.48	11.377	14.460	14.690
	48	16.723	14.725	13.918	14.152
**CD4**	12	3.188	4.057	8.932	8.085
	24	6.498	8.543	13.358	14.075
	48	11.285	8.0183	9.598	10.883
**CD8-α**	12	19.980	17.782	18.408	19.242
	24	17.020	16.900	20.567	18.510
	48	8.310	18.228	19.548	19.065
